# Case Report: Atropine-induced delirium in a dog

**DOI:** 10.3389/fvets.2025.1654764

**Published:** 2025-10-13

**Authors:** Samantha Fedotova, Heidi B. Kellihan, Lesley Smith, Kyle Bartholomew

**Affiliations:** ^1^Department of Medical Sciences, School of Veterinary Medicine, University of Wisconsin, Madison, WI, United States; ^2^Department of Surgical Sciences, School of Veterinary Medicine, University of Wisconsin, Madison, WI, United States

**Keywords:** canine, continuous rate infusion, psychosis, anticholinergic, cardiac, atrioventricularblock

## Abstract

A 6-year-old spayed female Goldendoodle was presented as a referral for bradycardia and collapse. An electrocardiogram revealed a high-grade second-degree atrioventricular block (HG2AVB) with atrial flutter. The patient was hospitalized overnight with the intention of pacemaker placement the following morning. Following bolus administration of atropine and lidocaine to rule out high vagal tone, the patient was placed on an atropine continuous rate infusion (CRI) to increase heart rate overnight. This resulted in an acute onset of suspected delirium, which lasted several hours following cessation of the CRI. The patient proceeded with pacemaker placement the following day. No further neurological events were noted at 1 year after pacemaker placement. Atropine-associated delirium should be considered in any patient where high cumulative doses of atropine are being considered.

## Case report

A 6-year-old spayed female Goldendoodle was presented to the cardiology service at the university of wisconsin veterinary teaching hospital as a referral for bradycardia and episodes of collapse. The patient had experienced four episodes of collapse over a 2-week period and was evaluated by their primary care veterinary facility. The owners reported that no neurological signs were noted at home. The patient’s prior medical history included sensitivity to flea and tick preventatives, manifested as a week-long episode of hyporexia following administration of an unknown brand. On presentation, the patient was noted to be bradycardic (heart rate [HR] 45–60 bpm) with an irregular rhythm. The rest of the physical examination was unremarkable. An electrocardiogram (ECG) was obtained with the patient in lateral recumbency, initially showing a heart rate of 100 bpm. As the patient relaxed, the ECG showed evidence of a high-grade second-degree atrioventricular block (HG2AVB) with a heart rate ranging from 20 to 31 bpm. Thoracic radiographs were performed and were unremarkable. Immediate referral to XXX was recommended for pacemaker placement.

## Diagnostic workup

On the afternoon of admission, the patient was presented to the cardiology service at XXX. On presentation, the patient was bright, alert, calm, and very friendly. Cardiovascular examination showed a heart rate of 100 bpm with an irregular rhythm consisting of intermittent long pauses. Intermittent grade I–II left apical systolic and right apical systolic murmurs were noted. An electrocardiogram confirmed atrial flutter with a high-grade second-degree atrioventricular block (HG2AVB) and a heart rate ranging from 25 bpm to 100 bpm. The echocardiogram showed mild four-chamber dilation, suspected to be secondary to chronic bradycardia, but was otherwise relatively unremarkable with no compromise of systolic function. The patient was hospitalized overnight and placed on telemetry with plans to proceed with pacemaker placement the following day.

It is common practice to rule out a potential vagally mediated first- or second-degree AV block with an atropine response test, as high vagal tone has been implicated in cases of AV block (1). Notably, high vagal tone has also been reported as a potential etiology for atrial flutter and atrial fibrillation due to increased heterogeneity within the atrium (2). Vagally mediated atrial fibrillation in canine patients has been experimentally and clinically converted to a normal sinus rhythm using lidocaine (3, 4). To rule out the potential high vagal tone as the etiology of the HG2AVB and atrial flutter, the patient was administered 0.04 mg/kg of atropine and 2 mg/kg of lidocaine intravenously two separate times 1 h apart. No ECG response was noted to the administered dose of atropine or lidocaine. Based on history, physical examination, and abdominal ultrasound, causes of increased vagal tone were ruled out.

## Treatment and outcome

While the patient did not convert to a sinus rhythm after being given atropine, given the evidence of delayed conduction through the AV node, an atropine CRI was initiated at 0.02 mg/kg/h in an attempt to increase the heart rate. The bradycardia continued to be progressive (30–40 bpm) after 1–2 h, and the atropine CRI was increased to 0.04 mg/kg/h. Approximately 4 h following the onset of the CRI, the patient exhibited an acute onset of suspected delirium. At that time, the patient had received approximately 3.6 mg of atropine (0.16 mg/kg) over 5 h, including the two bolus doses before the CRI. The patient became frantic and very anxious and was noted to begin loudly vocalizing, howling, and barking. The patient was removed from the kennel and began attempting to bite the individuals handling her. During the event, the patient urinated and defecated. Once the patient was restrained, she appeared to be dazed and demonstrated a lack of visual response. The atropine CRI was discontinued, and 2 mg (0.09 mg/kg) of butorphanol was administered IV. The patient continued to be mildly anxious and was whining overnight, but no further acute behavioral events occurred. The following morning, the patient was anxious and whining during interactions, but the physical examination was otherwise unchanged from the previous day.

The neurology service was consulted due to the episode of suspected delirium. A neurological examination illustrated mydriasis and reduced pupillary light response in both eyes, suspected to be secondary to the residual effects of atropine administration. The rest of the neurological examination was within normal limits. As a result, it was determined that the delirium episode did not have a primary neurological cause. Following the workup, the decision was made to proceed with transvenous pacemaker placement via a jugular venotomy under fluoroscopic guidance. A premedication of 0.1 mg (4mcg/kg) fentanyl and 2 mg (0.09 mg/kg) atropine was used, and 20 mg (0.9 mg/kg) of alfaxalone was used to induce anesthesia. The patient was maintained on a 5–10 mcg/kg/h fentanyl CRI and isoflurane. One dose of 0.88 mg (0.04 mg/kg) of atropine and 480 mg (21.8 mg/kg) of cefazolin was administered intraoperatively. The pacemaker placement was successful with no adverse events. The patient was given a low dose of 0.1 mg (4mcg/kg) of acepromazine prior to extubation. The patient recovered well but was profoundly sedate for an extended period of time following the procedure. The following day, the patient was hypersalivating but was not anxious during the physical examination. Previously noted mydriasis and reduced pupillary light reflexes had resolved. The follow-up physical examination remained unchanged compared to the previous one. The patient was discharged the following day. Since discharge, the patient was last seen 1 year following pacemaker placement, with no additional neurological events noted. MDR1 genetic testing was submitted and confirmed that the patient was negative for the MDR1 gene mutation.

## Discussion

Atropine, or atropine sulfate, is an alkaloid that originates from the plant *Atropa bella-donna* and is used for its antimuscarinic properties (5, 6). The use of *Atropa bella-donna*’s properties was first described in 1550 BC for the treatment of colic in children (5). Following the extraction of the alkaloids from their respective plant material, in 1875, Ringer published an in-depth handbook of the effects of atropine in his human patients. One of the reported effects was severe delirium, especially when patients were administered high doses of atropine (6). From the 1950s to the 1970s, a series of studies evaluated delirium caused by atropine toxicity as a potential therapy for multiple mental disorders (7). As a result, there are many descriptions in the human literature detailing the clinical manifestation of delirium from atropine toxicity. Experimentation with atropine in animals dates back to the early 19th and 20th centuries, during which multiple experiments were conducted in various animal species given high doses of atropine and monitored for its effects. At high doses, hyperexcitability, agitation, or a sedated coma state was described similarly across species, with variations in the dose required to elicit these effects (8).

Atropine’s mechanism of action is through the inhibition of the postganglionic muscarinic receptors and direct vagolytic action (6, 9). The muscarinic receptors M1–M4 exist as a heterogeneous population at various concentrations in different parts of the body, including the brain (10). Unlike other anticholinergics (i.e., glycopyrrolate), atropine can cross the blood–brain barrier and act on receptors located within the brain (6, 11). While there is no FDA approval for the use of atropine in canine patients, the standard dose for the atropine response test is 0.04 mg/kg intravenously or subcutaneously. In cases of organophosphate toxicity, multiple injections of 0.2 mg/kg of atropine are recommended while clinical signs persist (12). The most common adverse effects of atropine are related to exacerbation of anti-parasympathetic effects. These side effects include constipation, xerostomia, tachycardia, changes in vision, hyperthermia, and difficulty urinating. At high doses, delirium, excitability, agitation, and coma have been reported (6, 9). The dose required to induce delirium is species-dependent. There is little consensus on the toxic dose at which delirium occurs in animal species due to difficulty interpreting animals’ mental states. Syndromes involving excitability and depression, described as delirium, have been observed in canine species at doses starting at 1.5 mg/kg (8).

As previously mentioned, delirium is well-described in the human literature. At dosages of 10 mg/kg or higher, the described delirium consists of hyperactivity, incoherent speech, vocalizations, visual/auditory hallucinations, and confusion (5–8). In modern human medicine, there are descriptions of atropine-induced delirium following high doses of intravenous atropine (13, 14). Notably, there is also a case report that described atropine-induced delirium in an elderly man following the use of atropine eye drops (15). While bolus atropine dosing is more common, CRI administration of atropine combined with pralidoxime has been used in human medicine for the treatment of acute organophosphate toxicity and may be more effective (16). Additionally, in human medicine, a scale has been developed to objectively quantify the severity of atropine-induced delirium (10). Unlike human medicine, atropine-induced delirium in veterinary medicine is not well described, which may be due to the difficulty categorizing the nuances of mental status in veterinary patients. Experimentally, suspected delirium (hyperactivity and agitation) has been noted in various veterinary species such as cats, rats, monkeys, and dogs (8). Clinically, there is one report of an elephant exhibiting an episode of delirium following atropine administration before an anesthetic event (17). The patient in this case report illustrated evidence of agitation, excitability, and vocalization without an identified primary neurological abnormality. It was highly suspected that the acute neurological episode exhibited by this patient was a result of atropine-induced delirium. This is the first clinical case report in modern veterinary medicine of a canine patient exhibiting delirium because of high doses of atropine.

It is important to note that the patient received a total of 4 mg/kg of lidocaine intravenously at the same time as the administration of two bolus doses of atropine. Lidocaine has been associated with neurological signs such as muscle twitching, seizures, and coma (6). While we considered that lidocaine could have contributed to the altered mental state in the dog reported here, given the length of time between the administration of lidocaine and the onset of clinical signs of delirium during the administration of the atropine CRI, it seems more likely that it was the cumulative dose of atropine that contributed to the observed delirium in this patient.

The mechanism of delirium from high doses of atropine is not fully understood. The predominant theory is that atropine’s antagonism of the M1 receptor is involved in inducing delirium (10, 18). Many of atropine’s actions throughout the body are carried out through antagonism of the M1–M4 muscarinic receptors, which are present at different concentrations throughout different parts of the body. M1 receptors are located exclusively in the brain and are involved in perception, attention, and cognitive function (10). The peripheral effects of atropine are mediated by the M2–M4 receptors and are closely correlated to the plasma atropine concentrations (10, 18). The onset of the central effects is delayed from the time of peak plasma drug concentration. Similarly, the duration of the central effects is significantly longer than expected given the rapid plasma clearance of atropine (10). One study in humans illustrated that atropine’s effects on cognitive and behavioral tasks persisted over 7 h after peak drug concentrations in the plasma (18). One theory for the delayed onset and long duration of the central effects of atropine is the M1 receptor mechanism of action. In contrast to faster-acting M2–M4 receptors, M1 receptors have a slower onset and a longer duration (19). As a result, it is suspected that the central effects of atropine will have a mildly delayed onset and will persist long past peak plasma concentrations. The patient described in this case report, despite sedation, continued to exhibit signs of anxiety overnight and throughout the next morning several hours after stopping the atropine infusion. It is believed that the patient was experiencing the central effects of high-dose atropine despite not receiving any further doses of atropine for many hours. It is important to consider that the central effects of atropine-induced toxicity have the potential to last longer than the peripheral anticholinergic effects when considering high-dose administration.

The patient in this case report was presented with atrial flutter and HG2AVB, which have both been associated with high vagal tone (1, 2). The echocardiogram revealed no structural abnormalities. As a result, high vagal tone was considered a potential etiology to explain both atrial flutter and HG2AVB. To rule out high vagal tone, atropine and lidocaine were administered twice, at the same time an hour apart, to attempt to convert the patient to a sinus rhythm. Lidocaine is typically contraindicated in cases of HG2AVB due to repression of potentially life-saving ventricular escape complexes. However, given the consideration for vagal etiology and evidence of conduction through the AV node, an attempt was made to convert the patient to a normal sinus rhythm. Following these failed attempts to convert the underlying arrhythmia, the patient was placed on an atropine CRI to maintain an appropriate heart rate overnight. The cumulative dose of atropine is suspected to be the underlying reason for the patient’s resulting delirium. It is worth considering, however, that the cumulative dose (0.16 mg/kg IV) was within the dosage recommended to reverse cholinergic toxicity (0.2 mg/kg IV) and significantly below the dosage that experimentally induced instances of delirium in canine patients (5–10 mg/kg IV) (12). While this patient may have been sensitive to the drug, there is no published minimum dosage at which delirium occurs in dogs. It is worth noting then that high doses of atropine are not benign and can result in extended agitation and delirium in dogs.

The exhibited central effects of atropine may have been avoided through the selection of a different anticholinergic. For example, glycopyrrolate has been illustrated to have similar diagnostic potential in ruling out vagally mediated arrhythmias (11). Compared to atropine, glycopyrrolate has a slower onset by a few minutes and a longer duration of action by a few hours (12). Glycopyrrolate does not cross the blood–brain barrier; however, it has not been associated with central side effects (6, 11). As a result, glycopyrrolate could be considered a potential substitute for atropine when an anticholinergic is required in high doses. This should potentially be considered in any facet of veterinary medicine where multiple doses of atropine are being used or an atropine CRI is considered. In historic human literature, delirium post-anesthesia was attributed partially to the atropine used in anesthetic protocols (20). It is worth considering whether post-anesthetic dysphoria in canine patients could, in part, be attributed to serial atropine administration during procedures.

In summary, the clinical presentation of this patient is consistent with atropine-induced delirium. The perceived delirium lasted several hours past the last atropine administration, suggesting that the central effects persist past peripheral peak atropine concentrations. More research needs to be conducted to adequately categorize the potential for delirium in canine patients. However, given the potential for delirium, glycopyrrolate should be considered when exposing patients to high doses of anticholinergic medications.

## Data Availability

The raw data supporting the conclusions of this article will be made available by the authors, without undue reservation.
